# Complete Genome Sequences of *Xanthomonas axonopodis* pv. *glycines* Isolates from the United States and Thailand Reveal Conserved Transcription Activator-Like Effectors

**DOI:** 10.1093/gbe/evz085

**Published:** 2019-04-26

**Authors:** Sara C D Carpenter, Lawan Kladsuwan, Sang-Wook Han, Sutruedee Prathuangwong, Adam J Bogdanove

**Affiliations:** 1 Plant Pathology and Plant-Microbe Biology Section, School of Integrative Plant Science, Cornell University; 2 Department of Plant Pathology, Faculty of Agriculture, Kasetsart University, Bangkok, Thailand; 3 Department of Plant Science and Technology, Chung-Ang University, Anseong, Republic of Korea

**Keywords:** *Xanthomonas axonopodis* pv. *glycines*, *Xanthomonas citri* pv. *glycines*, TAL effectors, bacterial pustule of soybean, PacBio sequencing, plant pathogenic bacteria

## Abstract

To compare overall genome structure and transcription activator-like effector content, we completely sequenced *Xanthomonas axonopodis* pv. *glycines* strain 12-2, isolated in 1992 in Thailand, and strain EB08, isolated in 2008 in the United States (Iowa) using PacBio technology. We reassembled the genome sequence for a second US strain, 8ra, derived from a 1980 Iowa isolate, from existing PacBio reads. Despite geographic and temporal separation, the three genomes are highly syntenous, and their transcription activator-like effector repertoires are highly conserved.

## Introduction

*Xanthomonas axonopodis* pv. *glycines* (Xag) causes bacterial pustule of soybean, a disease characterized by small, yellow-to-brown, foliar lesions surrounded by chlorotic haloes that sometimes have a raised center (or “pustule”) caused by mesophyll cell hypertrophy (Groth and Braun 1986; Jones and Fett 1987). The lesions can coalesce into larger necrotic areas (Groth and Braun 1986), and the disease can cause defoliation, when severe, and yield loss (Hartman et al. 2015). Bacterial pustule occurs worldwide, but can be more problematic where the growing season is warm and wet, and when susceptible soybean varieties are grown (Swings and Civerolo 1993; Wrather et al. 2001).

Like other bacterial pathogens, Xag delivers, into the cells of its host, protein effectors that can aid in colonization and contribute to disease development. All examined strains of Xag encode transcription activator-like effectors (TALEs) (Kim et al. 2006; Park et al. 2008; Athinuwat et al. 2009; Chatnaparat et al. 2016; Seong et al. 2016), which localize to the host nucleus and activate host genes by binding to effector-specific promoter sequences (Doyle et al. 2013). TALE specificity is determined by a central region of helical repeats that form a superhelix around the DNA (Boch et al. 2009; Moscou and Bogdanove 2009; Deng et al. 2012; Mak et al. 2012). Each repeat binds a single, specific nucleotide by virtue of a polymorphic pair of neighboring amino acids called the repeat-variable diresidue (RVD).

TALEs are critical for virulence in several *Xanthomonas* that infect other plant species (Kay et al. 2007; Streubel et al. 2013; Cohn et al. 2014; Cox et al. 2017), activating key host susceptibility genes; allellic variation that prevents TALE binding and activation of an important susceptibility gene can confer genetically recessive resistance to the pathogen through loss of susceptibility (Hutin et al. 2015). Depending on the host genotype, some TALEs may trigger plant defense by activating an executor resistance gene (Zhang et al. 2015). Such genes are genetically dominant. In the Xag-soybean pathosystem, some TALEs that trigger resistance have been identified (Park et al. 2008; Athinuwat et al. 2009), but the corresponding resistance genes have not. As of yet, no TALE has been shown to promote the development of bacterial pustule, but the recessive nature of the resistance gene *rxp* (Groth and Braun 1986; Kim et al. 2010) suggests the possibility: *rxp* could be an allele of a TALE-targeted susceptibility gene.

Although there is genome sequence available for two strains of Xag, 12-2 and 8ra (Chatnaparat et al. 2012; Seong et al. 2016), a TALE-encoding plasmid from a third strain has been sequenced (Kim et al. 2006), and a TALE-encoding (*tal*) gene was cloned and sequenced from another (Athinuwat et al. 2009), the distribution and diversity of TALEs in Xag is unknown, and the genetic diversity of the pathovar across different geographical regions is not well studied. Strain 12-2, isolated from a pustule on soybean cultivar SJ4 in 1992 in Nakornratchasima, Thailand (Thowthampitak et al. 2008), was sequenced using Illumina short-read sequencing (Chatnaparat et al. 2012), but Illumina sequencing does not yield a complete, contiguous genome or capture the repetitive TALE-encoding (*tal*) gene sequences. However, expression of at least one *tal* gene transcript by this strain was revealed by global, short-read RNA sequencing (Chatnaparat et al. 2016). Strain 8ra, a rifampin-resistant derivative of strain Xcg-8, isolated from a symptomatic soybean plant (cv. BSR301) in Iowa in 1980 (Groth and Braun 1989), was sequenced by Seong et al. (2016) using PacBio, long-read technology, but the accuracy and completeness of the *tal* genes in the final assembly are uncertain. PacBio generally captures *tal* genes well, but the standard approach to assembly of PacBio data can sometimes result in errors in *tal* gene assembly. These can be corrected by preassembly of *tal* gene sequence-containing reads (Booher et al. 2015). This preassembly approach was not used for 8ra.

Identification of the complete sets of TALEs in different isolates can be an important first step toward development and targeted regional deployment of resistant soybean varieties, and comparison of whole genome structure across strains can yield insight into the overall genetic diversity of the pathogen. For this purpose, in order to determine and compare their TALEs and genome structures, we resequenced strain 12-2 using PacBio technology to generate a complete, contiguous assembly including any and all *tal* genes, and we reassembled the 8ra genome using the previously generated PacBio reads to reassess *tal* gene content. Additionally, we used PacBio sequencing to generate a complete, *tal* gene-replete assembly of a second US strain, EB08, which was isolated from soybean leaves collected in central Iowa in 2008 (Hummel et al. 2012). Note that accessions of the new assemblies as well as all previous Xag sequences in GenBank are annotated as sequences of *Xanthomonascitri* pv. *glycines*, per NCBI policy, following a recent suggested reclassification (Constantin et al. 2016).

## Materials and Methods

Strains 12-2 and EB08 were cultured, genomic DNA was isolated, and 20 kb genomic libraries were prepared and size-selected as previously described (Booher et al. 2015). Each library was sequenced using two SMRT cells on a PacBio RSII machine, which yielded 145× coverage for 12-2 and 230× for EB08.

The EB08 and 12-2 genomes were assembled using the HGAP assembler version 2.0, the 8ra genome was assembled using version 3.0, and all were manually circularized and polished twice more with Quiver (Chin et al. 2013) as described (Booher et al. 2015). The *tal* genes were preassembled and verified using PBX, and EB08 and 12-2 *tal* gene content was additionally verified by Southern blot, as previously described (Booher et al. 2015).

The whole genome assemblies were verified using PBHoney (PBSuite 14.7.14; English et al. 2012). This showed a 36-kb excision in a portion of the reads for 12-2, predicted by PHAST (Zhou et al. 2015) to be a prophage; this was retained in the final assembly. Since size-selection of long reads can exclude extrachromosomal DNA smaller than 7 kb, we checked EB08 and 12-2 for small plasmids using the Qiagen HiSpeed Midi Kit (Qiagen) and gel electrophoresis, which revealed a small plasmid of 1.8 kb in both strains. These small plasmids were sequenced by primer walking and Sanger sequencing to a depth of at least 2×, using initial sequencing primer (B2207, 5′-CCCGGGGTGTGGGGC), designed based on sequence of pXAG82 (Kim et al. 2006), another small *Xanthomonas* plasmid.

## Genome Structures for 12-2, EB08, and 8ra Are Similar

The genomes of 12-2 and EB08 each include a ∼5 Mb chromosome (5,398,926 bp in 12-2 and 5,333,566 bp in EB08), a 26.6-, and a 1.8-kb plasmid. The new assembly of 8ra yields a chromosome of 5,364,156 bp, and the same 26.6 kb plasmid, previously named pXAG81 (Kim et al. 2006) and pXAG_1 (Seong et al. 2016). The data do not capture the small plasmid of this strain, pXAG82, previously sequenced by Kim and colleagues (Kim et al. 2006). The shift in chromosome size for 8ra (from 5,363,581 bp) is due in part to revision of the sequence of *tal1a*, which had been incorrectly assembled as a pseudogene originally, but is in fact an intact gene. The plasmids of 12-2 and EB08 were named, and the large plasmid of 8ra renamed, by size in the convention established by Thieme and colleagues (Thieme et al. 2005) and by strain, for example, pXAG27.12-2, pXAG27.EB08, pXAG2.12-2, etc. The large plasmids in each strain are 99% identical to one another, and 99% identical to a 10.7 kb segment of pAG1, a 15.1 kb plasmid from strain AG (Kim et al. 2006). pXAG27 carries likely *mob* genes and may be transmissible. The small plasmid, pXAG2, is identical between 12-2 and EB08 except for a single synonymous mutation in an open reading frame similar to the putative replication protein on pXG31 (Baldini et al. 1999). pXG31 is the closest BLAST hit to pXAG2. The small plasmid in 8ra is 1.3 kb and is not similar to pXAG2.

**Fig. 1.**
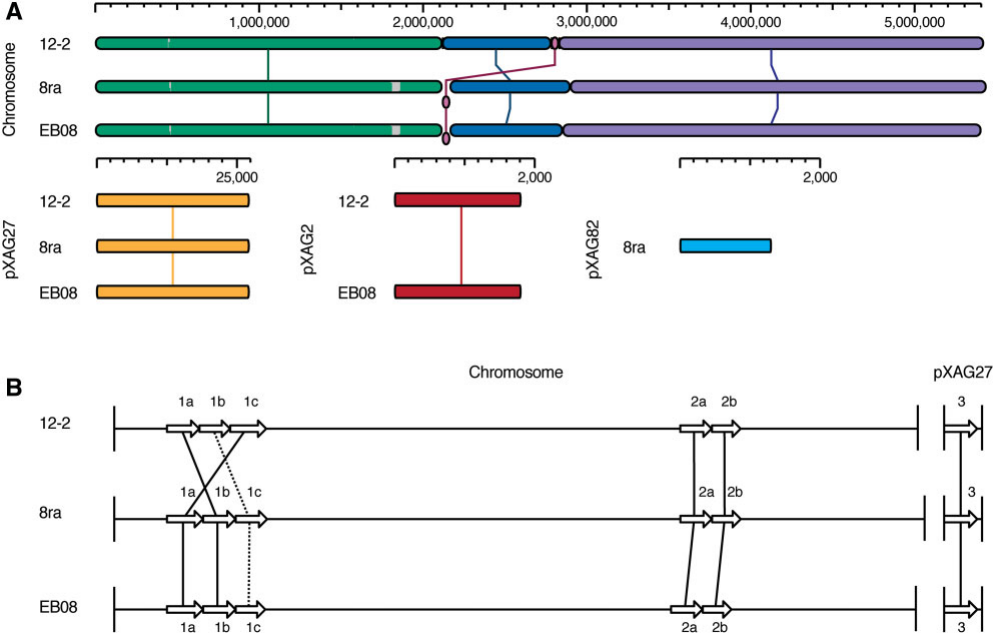
—Comparison of whole genomes and *tal* genes of 12-2, EB08, and 8ra. (A) Alignment of the 12-2, EB08, and 8ra genomes, generated with MegAlign Pro v.15.3 (DNASTAR, Madison, NJ) using progressiveMAUVE. Chromosomes and plasmids are shown linearized. Horizontal axes show sequence coordinates (bp). Matching colors represent locally colinear (syntenic) blocks. (B) The *tal* genes of strains 12-2, EB08, and 8ra. The genes are represented as white arrows at their relative positions in the linearized chromosome or plasmid, not to scale. Solid lines connecting two arrows indicate RVD sequences with one or no substitutions. Dashed lines connecting two arrows indicate RVD sequences with one or more RVD deletions. For label clarity, *tal* gene names are shortened to number/letter (e.g., *tal1a* becomes 1a).

ProgressiveMauve (Darling et al. 2010) alignment of 12-2, EB08, and the new assembly of 8ra (fig. 1*A*) shows the same chromosomal arrangement in the Iowan strains, despite 28 years between their isolation. Surprisingly, the chromosomal organization of Thai strain 12-2 is largely similar to that of the Iowan strains, except that a 47 kb region in the chromosome has been transposed and is 49 kb in 12-2. This chromosomal stability is in stark contrast to the genomic plasticity of *Xanthomonas oryzae*, and may be due to the presence of fewer IS elements in Xag than in *X. oryzae* (Bogdanove et al. 2011).

## The TALE-Encoding Genes of 12-2, EB08, and 8ra Are Well Conserved

**Fig. 2.**
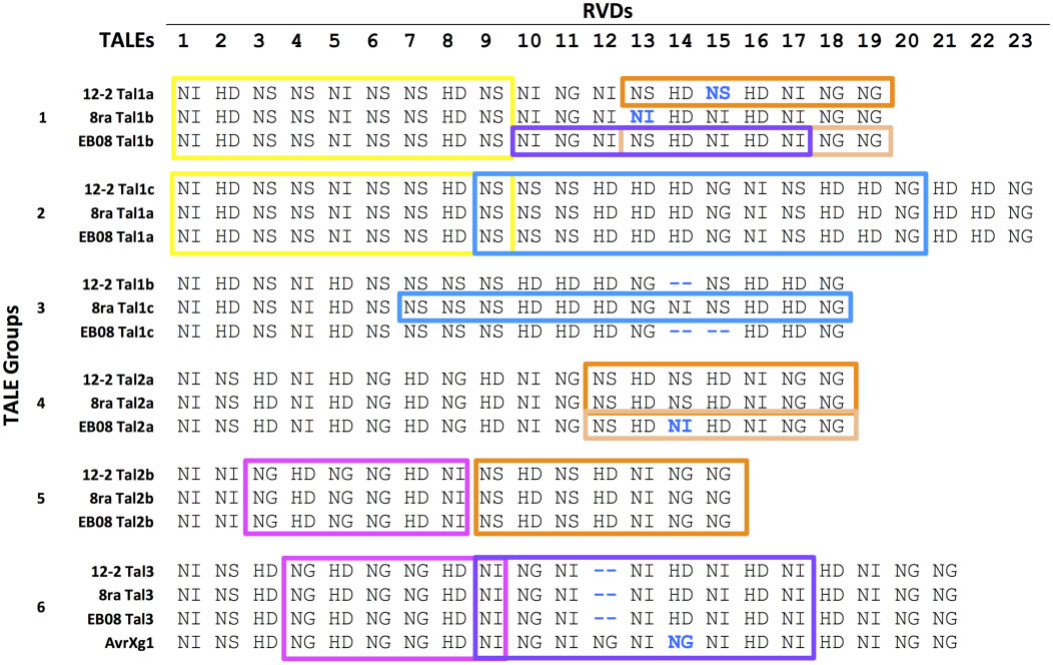
—Alignment of RVDs of TALEs of Xag strains 12-2, EB08, and 8ra. RVDs that differ between otherwise highly conserved TALEs are in bold, blue font. Strings of RVD sequences shared between two TALE groups are outlined in like color boxes.

EB08, 12-2, and 8ra each encode six TALEs all perfectly or nearly perfectly conserved across strains, forming six orthologous groups (figs. 1*B* and 2). Five are encoded on the chromosome in two clusters, and one (*tal3*) is encoded on the plasmid pXAG27, which, as noted, may enable conjugal transfer. The arrangement of the *tal* genes is the same in the two Iowan strains, and nearly so across all three strains, with only a transposition of two genes in the *tal1* cluster in the Thai strain relative to the Iowan strains (fig. 1*B*).

Orthologous TALE groups 2 (Tal1c_12-2_/Tal1a_8ra/EB08_), 5 (Tal2b), and 6 (Tal3) have completely conserved RVD sequences (fig. 2). The previously published Xag TALE AvrXg1 (Athinuwat et al. 2009) closely matches group 6, with one extra repeat and one RVD substitution (fig. 2). The other three groups have one or two RVD differences within them. Blocks of RVD sequences are also very similar between groups (fig. 2), suggesting that new *tal* genes may have formed by duplication and recombination in an ancestral strain. For example, a string of 9 RVDs present in group 1 TALEs (fig. 2, yellow highlight) and a string of 12 RVDs in the 8ra group 3 TALE (blue highlight) make up the majority of the RVD sequence of TALEs in group 2.

Given the high similarity among *tal* gene sequences generally, and their repetitive nature, a greater degree of diversity than observed in *tal* gene content across the three Xag strains might be expected due to recombination. That the *tal* effector content has been relatively static over nearly three decades and across two continents is thus surprising. The conservation of these TALEs over time and space may indicate that they perform a critical function, and if so, that there is little to no genetic variation at their targets across commonly grown soybean varieties, such that there is no selective pressure on the *tal* genes to adapt. If TALEs are similarly conserved in a wider sampling of Xag strains, and evidence of a role in pathogenicity emerges, Xag TALEs may make good targets for the development of resistance, either by engineering an executor resistance gene to be activated by one or more of the TALEs or by editing the TALE virulence targets to prevent activation.

## Conclusion

We generated whole genome sequences of Xag strains EB08 and 8ra from the United States and 12-2 from Thailand to examine their relatedness and TALE content and found them to be surprisingly similar. Despite decades of time and origins half a world apart, the genomes of the three strains are similarly arranged, and the TALEs are largely conserved, the latter of which may indicate stationary host genetic targets that might be modified to develop durable resistance. Use of long-read sequencing to capture the genomes of an even greater sampling of diverse Xag isolates will advance understanding of Xag evolution, help identify TALEs and possibly other effectors under selection as virulence determinants, and aid in the development of effective, effector-targeted means of resistance.

## Notes

**Data deposition:** This project has been deposited at GenBank under the accessions PRJNA323439 for strain 12-2, PRJNA431457 for strain EB08, and PRJNA341902 for strain 8ra.
